# Scribble basal polarity acquisition in RPE cells and its mislocalization in a pathological AMD-like model

**DOI:** 10.3389/fnana.2022.983151

**Published:** 2022-09-23

**Authors:** Alicia Segurado, Alba Rodríguez-Carrillo, Bárbara Castellanos, Emiliano Hernández-Galilea, Almudena Velasco, Concepción Lillo

**Affiliations:** ^1^Department of Cell Biology and Pathology, University of Salamanca, Salamanca, Spain; ^2^Institute of Neurosciences of Castilla y León (INCyL), University of Salamanca, Salamanca, Spain; ^3^Plasticity, Degeneration, and Regeneration of the Visual System Group, Institute for Biomedical Research of Salamanca (IBSAL), Salamanca, Spain; ^4^Department of Surgery, Ophthalmology Service, University Hospital of Salamanca, University of Salamanca, Salamanca, Spain

**Keywords:** Scribble, cell polarity, epithelium, differentiation, retina, retinal pigment epithelium (RPE), Scribble complex, age-related macular degeneration (AMD)

## Abstract

Apicobasal polarity is a hallmark of retinal pigment epithelium cells and is required to perform their functions; however, the precise roles of the different proteins that execute polarity are still poorly understood. Here, we have studied the expression and location of Scribble, the core member of the polarity basal protein complex in epithelial-derived cells, in human and mouse RPE cells in both control and pathological conditions. We found that Scribble specifically localizes at the basolateral membrane of mouse and human RPE cells. In addition, we observed an increase in the expression of Scribble during human RPE development in culture, while it acquires a well-defined basolateral pattern as this process is completed. Finally, the expression and location of Scribble were analyzed in human RPE cells in experimental conditions that mimic the toxic environment suffered by these cells during AMD development and found an increase in Scribble expression in cells that develop a pathological phenotype, suggesting that the protein could be altered in cells under stress conditions, as occurs in AMD. Together, our results demonstrate, for the first time, that Scribble is expressed in both human and mouse RPE and is localized at the basolateral membrane in mature cells. Furthermore, Scribble shows impaired expression and location in RPE cells in pathological conditions, suggesting a possible role for this protein in the development of pathologies, such as AMD.

## Introduction

Cell polarity is achieved thanks to the coordinated functions of three protein complexes that are highly conserved in evolution: the Crb and Par complexes (in the apical domain of cells) and the Scribble complex (in the basal domain) (Assémat et al., 2008; Rodriguez-Boulan and Macara, 2014). The correct acquisition of cell polarity is essential for specific cell and tissue functions (Assémat et al., 2008; Kaplan et al., 2009). The Scribble gene (and the protein) was first identified in *D. melanogaster*, where it was described as a crucial regulator of morphogenesis. It was also identified that mutations in Scribble cause drastic defects in the epithelial organization, resulting in rounded, irregularly shaped cells. In these models, it was also described that Scribble exerted its function by confining the localization of the Crb complex to the apical zone of the cell, excluding it from the basolateral domain and assisting in the correct establishment of the cell-cell adherens junctions (Bilder and Perrimon, 2000; Bilder et al., 2000; Assémat et al., 2008). The mammalian Scribble complex is composed of three proteins, Scribble *(Scribble planar cell polarity protein)*, DLG *(Discs large MAGUK scaffold protein)*, and LLGL *(LLGL scribble cell polarity complex component)*, and it is associated with the basolateral domain of the epithelial cell membrane, overlapping adherens junctions (Navarro et al., 2005; Qin et al., 2005; Kallay et al., 2006; Humbert et al., 2008). Scribble complex is expressed in numerous mammalian tissues and cell types, although the highest expression levels have been found in epithelial cells (Navarro et al., 2005). Low levels of these proteins have been described in the kidney, skeletal muscle, liver, and lung, while the highest levels are in the intestine, breast, placenta, and skin (Navarro et al., 2005; Assémat et al., 2008; Su et al., 2013). This protein complex has also been localized in the developing mouse retina (Nguyen et al., 2005), the epithelium of the human colon (Gardiol et al., 2006), and it even participates in fundamental processes of the immune response (Barreda et al., 2020). These proteins function cooperatively to establish and regulate cell polarity, junction formation, cell growth, and migration in most epithelial cells (Kallay et al., 2006; Yamanaka and Ohno, 2008; Su et al., 2012).

Malfunctions in the Scribble complex are associated with severe alterations in cell polarity. For example, during development in mammals, point mutations cause the mislocalization of the protein, resulting in perinatal death due to defects in neural tube closure (Robinson et al., 2012; Lei et al., 2013). Mislocalization or loss of Scribble disrupts polarity in human epithelial cells, leading to dysregulation in their function and contributing to tumor development; although they play a role as tumor suppressors in certain epithelial cancers, how Scribble contributes to this anomaly is not yet well-understood (Zhan et al., 2008; Pearson et al., 2011; Feigin et al., 2014; Liu et al., 2017). Furthermore, it causes a delay in tight junction formation and impaired E- cadherin recruitment to the membrane and, as a result, cells undergo an epithelial-mesenchymal transition process (Qin et al., 2005; Yamanaka and Ohno, 2008; Ivanov et al., 2010).

The retinal pigment epithelium (RPE) is a monolayer of epithelial cells that exhibits a marked apicobasal polarity (Bok, 1993; Rizzolo, 1997; Strauss, 2005; Lehmann et al., 2014). The integrity and maintenance of its functions depend on the establishment of this polarity and cell-cell junctions (Rahner et al., 2004; Rizzolo, 2007). As in other epithelial cells, these processes are functionally coupled due to the presence and correct functioning of the polarity complexes. Apical complexes, Par and Crb, have been described in the retina and in the RPE cells (van de Pavert et al., 2004; Luo et al., 2006; Park et al., 2011; Paniagua et al., 2015, 2021), but the expression and localization of the Scribble complex remain largely unexplored. Very few works have dealt with the localization and functions of this complex in the RPE cells. One of them has focused on the expression of the proteins Scribble, DLG, and some adhesion proteins in ocular tissues during the development of the mouse eyecup, particularly on the iris and the cornea (Nguyen et al., 2005). Another one has analyzed the alterations in the localization and functions of some members of the Scribble complex in a model of ocular adenocarcinoma. Both the mislocalization and downregulation of DLG, Scribble, and LLGL proteins seem to be correlated to tumor progression in this mouse model of adenocarcinoma (Vieira et al., 2008).

Then, all these previous works in different epithelial-derived tissues, including the retina, have suggested that the Scribble complex is not only involved in the establishment of the basal domain during development and mature conditions, but its alterations in pathological conditions could also be closely related to a starting point in the progression of diseases depending on the maintenance of apicobasal polarity. Nevertheless, this hypothesis still needs to be deeply explored. In addition, the involvement of the polarity proteins, especially those of the Scribble complex, in aging processes, such as Age-Related Macular Degeneration (AMD), where RPE cell barrier maintenance is compromised, is still largely unknown. Due to the critical nature of polarized pathways in RPE functionality, any anomalies in the organization and/or maintenance of the cell-cell junctions will have a major impact on the capability of the RPE to maintain choriocapillaris and photoreceptors. Moreover, in aging and AMD, these capabilities are compromised.

To better understand these processes, in the present work, we have first analyzed the time-course expression of Scribble in human RPE cells in culture, focusing on the expression and localization of this protein and others related to the polarization of these cells in the different apicobasal compartments over time. We observed not only an increase in the amount of Scribble during RPE development but also the fact that Scribble localizes early at the basal domain during RPE development and gradually acquires a well-defined basolateral pattern as the tissue reaches a fully mature phenotype. Furthermore, the expression of Scribble was compared with that of other elements involved in RPE cell polarity establishment and cell junctions, both in human RPE cells in culture and in tissue-fixed mouse RPE cells, allowing us to establish its precise location in the basolateral membrane of the cells, along with the proteins that define RPE differentiation. Finally, to better understand the potential implications and modifications of Scribble in pathological conditions of RPE cells, we have analyzed the role of this protein in human RPE cells subjected to experimental conditions that mimic the toxic environment that these cells undergo during the development of AMD. We observed an increase in Scribble expression in those cells, which develop a pathological phenotype, suggesting that this protein could be altered in situations of cellular stress, as occurs in AMD. This experimental model was established by exposing fully differentiated human RPE cells in culture to different concentrations of blood serum obtained from patients with atrophic AMD, exudative AMD, or control in a different timely manner. The results show that in these pathological conditions, both the expression and localization of Scribble get altered from the very beginning of the process, suggesting, as it has been shown in other epithelial tissues anomalies, a possible role in the onset and progress of aging pathologies, such as AMD.

## Materials and methods

### Animals

All procedures used in this work were in accordance with the guidelines of the European Directive 2010/63/UE and the RD 53/2013 Spanish legislation for the use and care of animals. All details of the study were approved by the Bioethics Committee of the University of Salamanca. For this study, seven 90-day-old adult wild-type C57BL/6J mice were used for *in vivo* immunofluorescence analysis. Animals were euthanized with carbon dioxide before tissue extraction.

### Human retinal pigment epithelium cell cultures (hRPE)

For *in vitro* studies, human RPE cells (LONZA) were used in the fourth passage, seeded at a density of 50000 cells/cm^2^ on Transwell^®^ polyester membrane inserts with 6.5 mm diameter and 0.4 μm pore size (Corning^®^) and maintained in RtEBM^®^ Basal Medium (LONZA), supplemented with L-Glutamine, FGF-B, and Gentamicin and Amphotericin-B. During the first 3 days, 2.5% fetal bovine serum (FBS) was also included. The medium was replaced every 3–4 days. To study the Scribble protein expression during RPE cell development and maturation, samples at three different time points of culture were collected: 7, 14, and 21 days in culture (DIC). Subsequently, to study the expression of this protein in pathological conditions, once cells reached 21 DIC, they were incubated with the different types of blood serum obtained from patients with ophthalmological problems.

### Human serum samples

All studies have been done in accordance with the Code of Ethics of the World Medical Association (Declaration of Helsinki) for experiments involving humans. Blood serum samples from voluntary patients with atrophic or exudative AMD and control, obtained from the Ophthalmology Service of the University Hospital of Salamanca, were used in this study. The use of human samples was approved by the Clinical Research Ethics Committee of the Institute for Biomedical Research of Salamanca (IBSAL) (PI15/01240; PI18/01536), the Drug Research Ethics Committee of the Regional Ministry of Health of the Junta de Castilla y León, and all the participants signed written informed consent. Clinical evaluation, diagnosis, and the subsequent recruitment of patients were carried out by ophthalmologists from the same hospital service. To be included in the study, patients had to meet the following criteria: (i) age >50 years; (ii) newly diagnosed with AMD; (iii) no previous treatment for AMD; (iv) no abnormalities that could interfere with the study and/or other degenerative diseases. Control sera were obtained from healthy volunteers, based on the same inclusion criteria. Therefore, patients were classified into three groups: (1) *Atrophic or dry AMD*: patients with intermediate, extensive, or large macular drusen (≥125 μm diameter); (2) *Exudative or wet AMD*: patients with choroidal neovascularization and/or its clinical manifestations (e.g., subretinal hemorrhages, retinal pigment epithelium detachments due to subretinal fluid accumulation); (3) *Control*: patients without AMD or any other macular disorder that could confuse the diagnosis. Blood samples were collected from patients at the time of diagnosis, before any treatment. After coagulation, serum was recovered by centrifugation, aliquoted in cryovials, and stored at −80°C until required.

### Exposure to human sera

Five serum samples from each group (5 atrophic or dry AMD, 5 exudative or wet AMD, and 5 controls) were used for this study. Briefly, mature confluent hRPE cells cultured in 24-well plates were exposed to different concentrations of the three types of blood serum and in a different timely manner. Separate plates were used for each type of serum, i.e., 5 24-well plates for serum from control patients, 5 for dry AMD serum, and 5 for wet AMD serum. Different concentrations of serum (5 and 10%) were diluted in the usual culture medium. This serum-medium mixture was added to the lower chamber of the Transwell^®^. 21 DIC cells were culture in this mixture for 3 and 7 days (DWS, *Days with serum*). Cells at the same stage of development, 24 and 28 DIC, respectively, with no serum added were used as a control for each period analyzed (non-serum group in results). Half of each plate (12 wells) was used for immunofluorescence experiments and the other half for western blot analysis. From this, half of the wells were analyzed at 3 days (DWS) and the other half at 7 days (DWS).

### Immunofluorescence labeling

The hRPE cells were fixed with 4% paraformaldehyde (PFA) for 10 min at 4°C. Then, were rinsed in PBS and stored at 4°C until use. For retinal cryosections, eyes were dissected and fixed by immersion for 30 min at 4°C in a solution containing 4% PFA in 0.1 M phosphate buffer at pH 7.4 (PB). The cornea and lens were then removed; retina, RPE, and choroid were further fixed by immersion for two additional hours at 4°C in the same fixative solution and washed in PB. Eyeballs were then cryoprotected in a graded sucrose series, embedded in Tissue-Tek O.C.T (Sakura), and stored at −20°C until sectioning. 14-μm tissue sections were obtained with a cryostat (Microm HM560, Thermo Fisher Scientific) and placed onto Superfrost Ultra Plus^®^ (ThermoFisher Scientific) slides and stored at −20°C until their use. Tissue autofluorescence was quenched with 0.25% sodium borohydride in 0.1 M phosphate-buffered saline, pH 7.4 (PBS) for 10 min at room temperature (RT). Both cells and tissues were then permeabilized in PBS with 0.02% or 0.1% Triton X-100 (PBS-Tx), respectively, and blocked for 1 h in 1% bovine serum albumin (BSA) and 5 % normal serum in PBS-Tx. Next, they were incubated overnight at 4°C with 1% BSA, 2% normal serum in PBS-Tx, and the primary antibodies for Scribble (Santa Cruz Biotechnology, 1:200), CRB2 [custom-made (Paniagua et al., 2015), 2.25 μg/ml], PAR3 (Millipore, 1:250), E-cadherin (Santa Cruz Biotechnology, 1:100), Occludin (Invitrogen, 1:200), and Apo-E (Millipore, 1:200). Subsequently, samples were washed with PBS-Tx and incubated for 1 h at room temperature with 1:750 Alexa fluor-488 and/or Alexa fluor-555 fluorescent secondary antibodies (Life Technologies), and/or Faloidin-FITC (Sigma, 1:500), and the nuclear marker DAPI (Sigma-Aldrich, 1:10,000) in 1% BSA and 2% normal serum in PBS-Tx. Finally, they were washed with PBS-Tx and PBS. Both cells and tissues were cover-mounted using Prolong Gold antifading reagent (Life Technologies) for subsequent microscopic analysis. Negative controls were also performed excluding primary and/or secondary antibodies in the incubation steps.

### Image acquisition and analysis

Images were obtained using an inverted Zeiss Axio Observer Z1 microscope (Carl Zeiss Microscopy, UC, USA) with a Zeiss Plan-Apochromat 40x NA 1.3 oil objective and processed with ZEN imagen software (Carl Zeiss Microscopy). To visualize the spatial arrangement of target proteins in cells, a z-stack of 20 images was taken for each field of view. Each set of images taken along the z-axis was transformed using a maximum sum algorithm into a single two-dimensional image called the maximum intensity orthogonal projection using Zen software. Images and projections optimized using Adobe Photoshop CS6 software and pseudo-colored for better comprehension with ImageJ software.

### Protein extraction and western blotting

Normal and serum-exposed hRPE cells were lysed in RIPA buffer (Bioscience) with a protease inhibitor cocktail (Sigma-Aldrich). Proteins were dissolved in Laemmli loading buffer and loaded onto an SDS-polyacrylamide gel under reducing conditions. After electrophoresis, proteins were transferred to PVDF membranes, blocked for 1 h at room temperature in Tris-buffered saline-Tween (0.1%) (TBST) with 2% BSA, and immunolabeled overnight at 4°C with primary antibodies for Scribble (Santa Cruz Biotechnology) and β-actin (Sigma-Aldrich) diluted in TBST solution with 2% BSA. After several washes in TBST, membranes were incubated with appropriate secondary antibodies in 5% non-fat dry milk in TBST for 1 h at RT. Membranes were then washed in TBST and developed with Clarity Western ECL Substrate (Bio-Rad) using a chemiluminescent imaging system (MicroChemi 4.2, Berthold Technologies). As negative controls, the PVDF membrane was incubated without the primary or secondary antibodies and both. Protein levels were measured by densitometric analysis using ImageJ software. The optical density obtained from each band was normalized against the corresponding β-actin and relativized vs. the negative control in all groups. Minor brightness adjustments were performed with Adobe Photoshop CS6.

### Statistical analysis

For each experiment, samples were obtained from individual wells, so for statistical purposes, each one is considered an independent sample. Half of each plate (12 wells) was used for IF experiments and the other half for WB analysis. From this, half of the wells were analyzed at 3 days (DWS) and the other half at 7 days (DWS). Statistical analyses were performed using Microsoft Excel (2016) (Microsoft Corporation), IBM SPSS Statistics software (version 20.0) (IBM Corp.; Armonk, NY, USA), and GraphPad Prism (version 8) (GraphPad Software, San Diego, California, USA). Samples were initially analyzed using the Kolmogorov-Smirnov test and Levene's test to check for normality of data distribution and homogeneity of variances, respectively. Statistically significant differences were analyzed by one-way ANOVA test followed by Bonferroni's *post-hoc* test in parametric data distribution, as well as the Kruskal–Wallis test, followed by Dunn-Bonferroni's *post-hoc* test was conducted for non-parametric data. A *p-value* of <0.05 (^*^) was considered statistically significant and <0.01 (^**^) highly significant. Data are given as mean ± standard deviation (SD). All experiments were performed independently and at least three times. The complete statistical analysis and data are included in Supplementary material.

## Results

### Scribble localizes at the basolateral membrane of human retinal pigment epithelium during development

**Figure 1:**
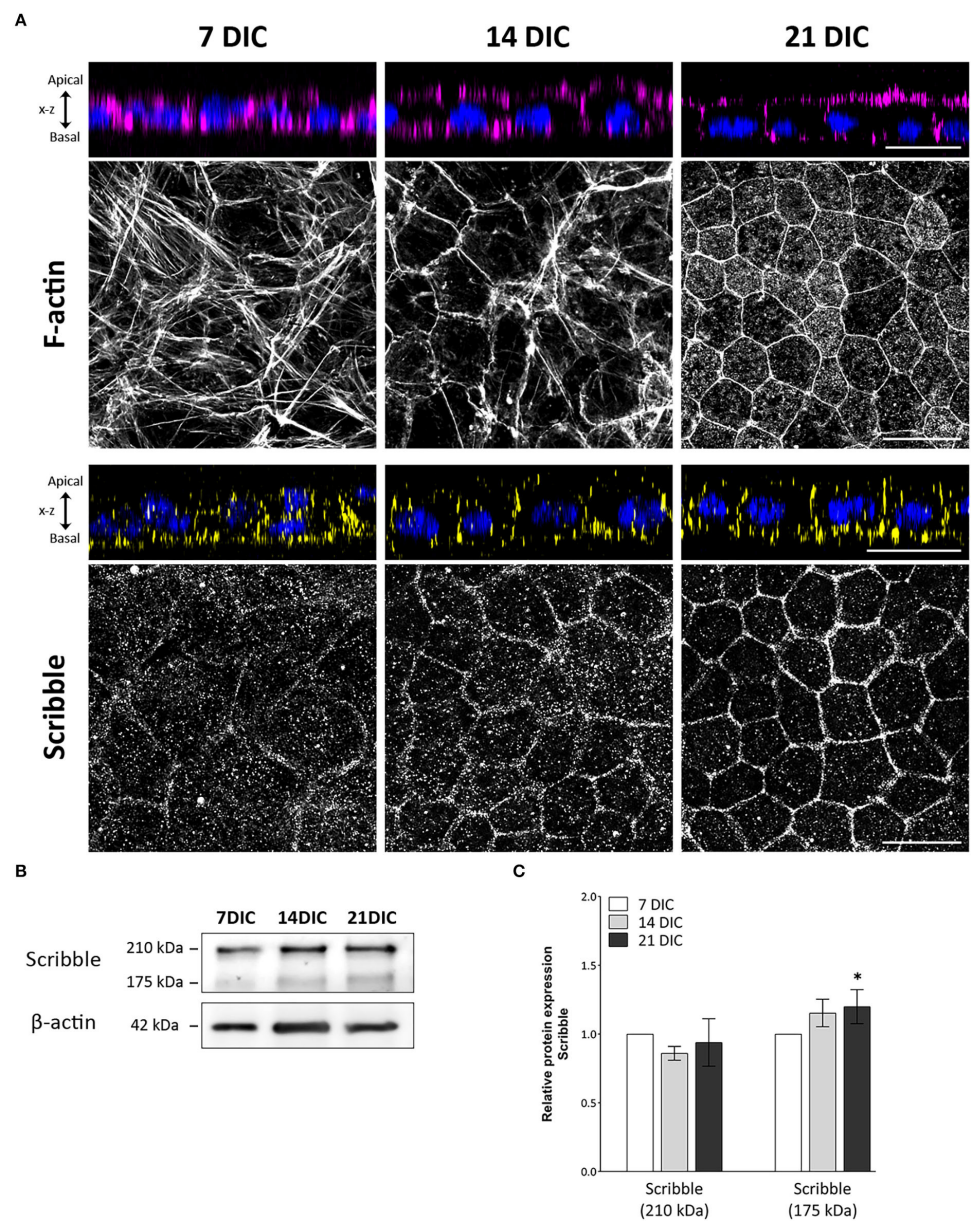
Time-course expression pattern of actin and Scribble protein during hRPE cell development in culture. **(A)** Orthogonal views and corresponding z-stack maximum projections showing actin (magenta) and Scribble protein (yellow) localization as hRPE cells gradually form the mature epithelial tissue. DAPI (blue): nuclear labeling. Scale bars: 20 μm. **(B)** Western blot analyses showing expression of Scribble (210 kDa) and Scribble (175 kDa) from 7 to 21 DIC. β-actin was used as loading control. **(C)** Graphical representation of the relative expression of Scribble (210 kDa) and Scribble (175 kDa) during the development of hRPE cells in culture. In both cases, 7 DIC (*n* = 9), 14 DIC (*n* = 9), and 21 DIC (*n* = 9). DIC in **(A–C)**:Days in culture. Data are presented as mean ± sd. *Statistical information*: The Kolmogorov-Smirnov and Levene tests were used to evaluate the normality and homoscedasticity of the sample distribution, respectively. Data were determined to be non-parametrically distributed. The potential significant statistical differences were analyzed by the Kruskal-Wallis test, followed by Dunn–Bonferroni's *post-hoc* test. Asterisks indicate statistical differences. *p-value < 0.05 *vs*. 7 DIC.

Well-defined circumferential actin belt formation is a determinant process in RPE cells for the acquisition of cell polarity (Miyoshi and Takai, 2008; Pfeffer and Philp, 2014). In these experiments, circumferential actin belt formation has been used then as a reliable indicator of the polarization rate of hRPE cells in culture. In this model, at 7 DIC, most actin filaments appeared as stress fibers. At 14 and especially at 21 DIC, a rearrangement of actin from stress fibers occurred, eventually resulting in a well-defined actin belt that precisely demarcates the cell periphery (Figure 1A). The three time points, 7, 14, and 21 DIC, were then established as checkpoints to define the developmental process of hRPE cells.

To elucidate the precise location of Scribble during the polarization and differentiation of human RPE cells, immunolabeling of hRPE cells during development in culture was performed. Axial and Z-stack images acquired along the Z-axis of immunolabeled hRPE cells in culture allowed us to precisely identify apical and basolateral areas of the cells (Figure 1A). Scribble expressed from day 7 in culture, when it displayed a punctuated pattern, was situated predominantly at the basal area, clearly outlining the irregularly shaped plasma membrane of some cells, which is a typical feature of early developmental stages. Scribble reorganized from the basal to the basolateral zone at 14 DIC. By this time point, there was a higher and more site-specific expression at the plasma membrane, although some cytoplasmic expression patches remained. At 21 DIC, Scribble showed a precisely defined localization in the basolateral domain of the plasma membrane, delineating all the neighboring cells. All hRPE cells at this time point exhibited a regular hexagonal shape, giving rise to the typical cobblestone pattern of a fully developed RPE monolayer. The expression of Scribble during maturation of hRPE cells in culture was also analyzed by western blot (Figure 1B). Scribble was detected at 210 and 175 kDa, two high-molecular weight bands that correspond to the two major isoforms of the Scribble protein produced by alternative splicing. The 175 kDa isoform is considered the canonical sequence. Analyzing this expression, both independently and altogether, Scribble appears from 7 DIC in hRPE cells, increasing from 7 to 21 DIC. The altogether expression of Scribble isoforms did not show statistically significant differences among 7, 14, and 21 DIC (*p* = 0.828; *n* = 27). However, when analyzing the two Scribble isoforms independently, although the 210 kDa isoform showed no significant differences among 7, 14, and 21 DIC (*p* = 0.112; *n* = 27), the 175 kDa isoform exhibited significant differences between 7 and 21 DIC (*p* = 0.016; *n* = 27) (Figure 1C). While the Scribble 210 kDa isoform remains constant throughout the developmental period, the Scribble 175 kDa isoform increases its expression during the maturation of the epithelial monolayer. Together, these data support the findings of the immunofluorescence analyses. Then, Scribble expression in hRPE cell culture increased during development up to 21 DIC and during this same period defined its basolateral localization.

### Scribble is located in the basolateral domain of the retinal pigment epithelium of the adult mouse

**Figure 2:**
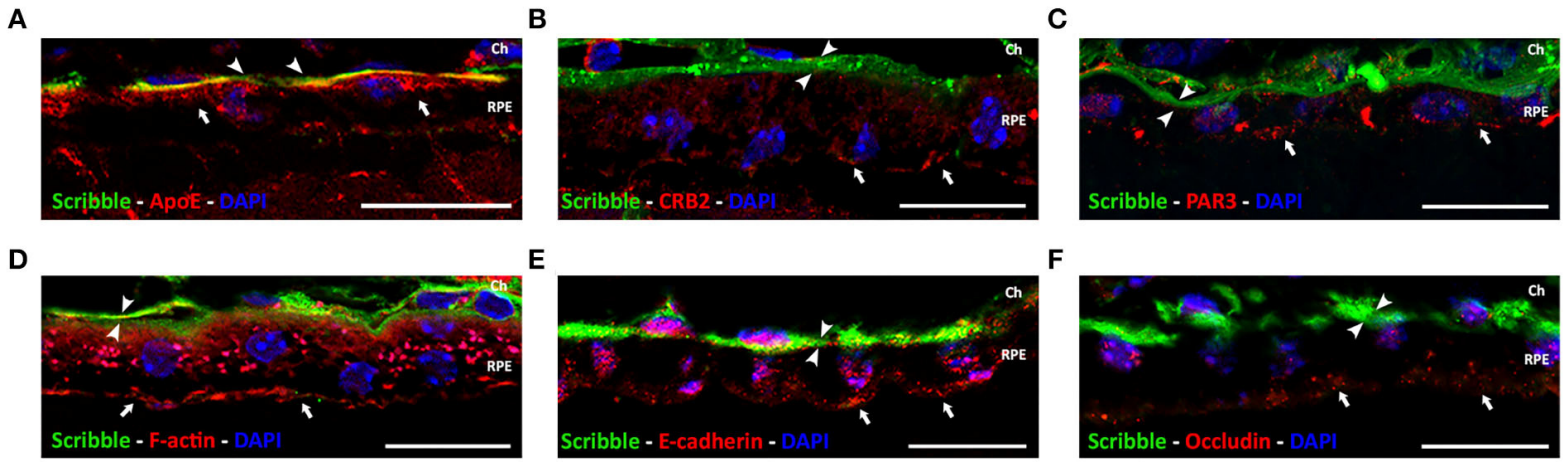
Localization of Scribble protein in wild-type adult mouse RPE. Double immunolabeling for Scribble (green, arrowheads) and other proteins (red, arrows): carrier protein for lipids, ApoE **(A)**; apical polarity proteins CRB2 **(B)** and PAR3 **(C)**; F-actin **(D)**; and adherens and tight junctional protein E-cadherin **(E)** and occludin **(F)**, respectively. DAPI (in blue): nuclear labeling. Scale bars: 20 μm. Ch, Choroid; RPE, retinal pigment epithelium.

Expression and localization of Scribble in tissue-fixed RPE of the adult wild-type mouse were determined by immunofluorescence. To define the precise subcellular localization of Scribble in this tissue, double immunofluorescence labeling was performed for Scribble and certain well-known proteins that perform specific functions in this cell type. To identify the basolateral domain of the RPE, the localization of Apolipoprotein E (ApoE), a carrier protein for lipids found in Bruch's membrane, was used as a marker. Double immunofluorescence for Scribble-ApoE showed that these two proteins are co-expressed in the same location, the basolateral domain of the RPE (Figure 2A). Similarly, apical domain-defining proteins that predominantly localize in this area were identified. CRB2 and PAR3 are both core components of the Crb and Par apical polarity complexes, respectively. Double immunofluorescence for Scribble-CRB2 (Figure 2B) and Scribble-PAR3 (Figure 2C) resulted in an opposed localization. Both CRB2 and PAR3 localized in the apical zone of the RPE cells, whereas Scribble defined their basolateral region (Figures 2B,C). In mature RPE cells, phalloidin stains the actin microfilaments that constitute the actin belt organized around the cell-cell adherens junctions. Scribble-Phalloidin double immunolabeling showed actin in an apical arrangement, whereas Scribble exhibited a basolateral localization (Figure 2D). Furthermore, the expression of E-cadherin (Figure 2E) and occludin (Figure 2F), two of the proteins involved in the establishment of adherens and tight junctions in the RPE cells, respectively, was also analyzed. As expected, since these proteins take part in the apical cell-cell junctions of the RPE cells, both were typically located in the apical domain in mature RPE cells, so the double labeling for Scribble and these two proteins highlighted the basolateral localization of the latter. Using this approach, it was possible to confirm that, in the mature and functional RPE, Scribble is expressed, localized, and conserved at the basolateral domain of the retinal pigment epithelium of the wild-type adult mouse.

### Effects of serum from patients with AMD on actin cytoskeleton integrity

**Figure 3:**
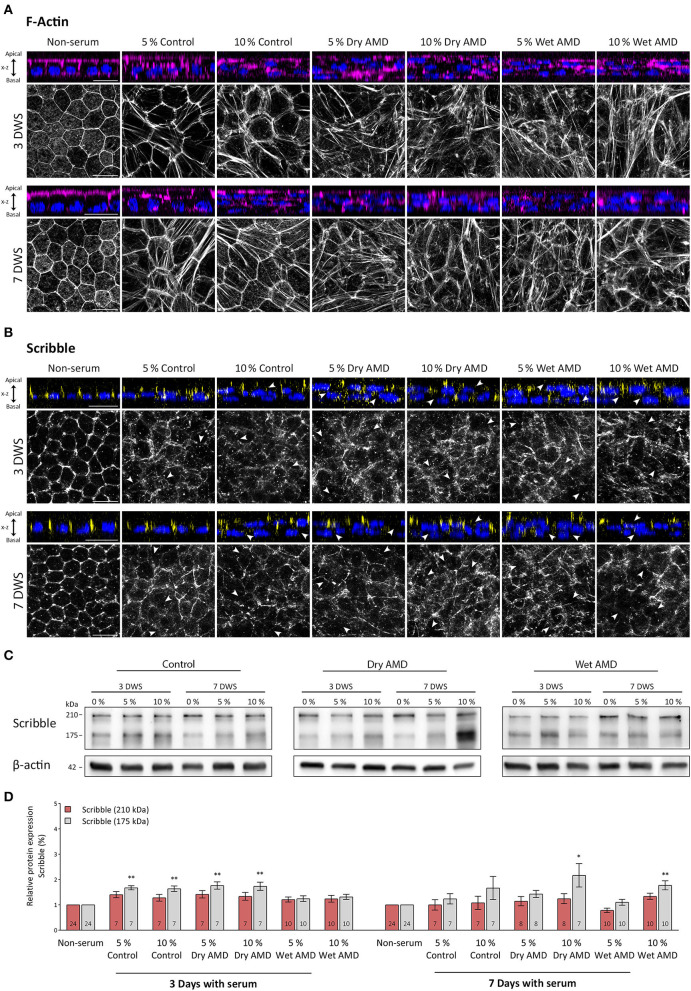
Expression pattern of actin and Scribble proteins in hRPE cells after serum exposure. **(A)** Orthogonal views and corresponding z-stack maximum projections showing actin (magenta) in cells without serum and after the serum exposure of both control patients and patients with AMD patients. **(B)** Orthogonal views and corresponding z-stack maximum projections showing Scribble (yellow) in cells without serum and after serum exposure of control patients and patients with AMD. DAPI (blue): nuclear labeling. Scale bars in **(A,B)**: 20 μm. **(C)** Western blot analyses showing expression of Scribble (210 kDa) and Scribble (175 kDa) in cells without serum and after control and AMD patients serum exposure. β-actin was used as loading control. DWS in **(A–C)**: Days with serum. **(D)** Graphical representation of the relative expression of Scribble (210 kDa) and Scribble (175 kDa) in cells without serum and after serum exposure of control patients and patients with AMD. Data are presented as mean ± SD. Arrows in **(B)** point out to areas where Scribble has translocated from the plasma membrane to the cytoplasm. The number within each column corresponds to the number of samples per data. Statistical information: The Kolmogorov-Smirnov and Levene tests were used to assess the normality and homoscedasticity of the sample distribution, respectively. Data were determined to be non-parametrically distributed. The potential significant statistical differences were analyzed by the Kruskal–Walli's test followed by Dunn–Bonferroni's *post-hoc* test. Asterisks indicate statistical differences. *p-value < 0.05; **p-value < 0.01 vs. non-serum group.

In this work, we have designed an experimental model to mimic, in hRPE cells in culture, the early modifications that RPE cells undergo in a toxic environment such as the early stages of AMD disease. To do this, hRPE cells, once differentiated and polarized at 21 DIC, were exposed basolaterally to serum obtained from patients with atrophic or exudative AMD or control as described in material and methods. The first characteristic analyzed was the organization of the circumferential actin belt. This structure is the most reliable indicator of the polarization state of hRPE cells and provides an insight of the strength and optimal conditions of cultured cells. One of the consequences of the exposure of hRPE cells to patients' blood serum was that actin cytoskeleton of hRPE cells was seriously altered (Figure 3A). Actin filaments of non-treated cells (non-serum) were arranged in the cell periphery, in a regular and well-defined ring, giving a mosaic-like appearance with all the pieces tightly fitting together (Figure 3A). Serum exposure caused a disruption of this organization, triggering an alteration of the cytoskeleton and the generation of stress fibers crossing the cytoplasm of the cells. Cells exposed to serum of control patients (5% and 10% control) showed an enlargement in cell size, as well as multiple stress fibers generation. These findings were more dramatic and visible in cells exposed to serum from patients with dry and especially wet AMD, where larger and more abundant stress fibers were observed (Figure 3A). Orthogonal views of the actin and nuclear immunostaining in the hRPE cells revealed that the monolayer of epithelial tissue observed in the non-treated hRPE cells transformed into a multilayered epithelial organization, especially visible in cells exposed to 10% of control serum and in any of the cultures exposed to serum from patients with dry or wet AMD (Figure 3A). In these cells, nuclei were often observed in clusters and in several layers, revealing that the typical epithelial monolayer of the mature RPE cells was completely disorganized after serum exposure. Spatial arrangement of the actin belt, located continuously in the apical zone of the untreated cells, was altered. In the cells exposed to patients' blood serum, actin labeling became patchy, cracked in appearance, and even disappeared in certain areas of the cell cultures. Stress fibers formation, the disorganized appearance, and the multilayered tissue transformation of RPE cells clearly demonstrated that serum exposure had an evident impact on the integrity of mature hRPE cells in culture. Such effects were observed in both control and patients with dry/wet AMD serum-exposed cells, although they were most prominent in the cultures exposed to serum of the two types of AMD.

### Scribble distribution in hRPE cells exposed to serum from patients with AMD

The effects of serum exposure on the expression and localization of Scribble in the hRPE cells were observed soon after this procedure since 3 days after the treatment, the distribution of Scribble was severely affected. In cells without human serum (non-serum), Scribble was found outlining the cell perimeter, showing a regularly well-defined appearance and the typical cobblestone pattern, while disorganization of this pattern was noticeable on serum-treated cells (Figure 3B). In all hRPE cell cultures treated with patients' blood serum, Scribble acquired a patchy pattern and showed a complete disarrangement from the cell membrane (arrows in Figure 3B). Only few patches of the protein remained, outlining the cells' structure. Although only few of the cells exposed to control serum retained Scribble expression in the plasma membrane in a diffuse and disorganized manner, the localization of Scribble (and the organization of the hRPE monolayer, as noted with F-actin labeling) was severely altered since Scribble was completely absent and displaced from the plasma membrane in these serum-treated cells. On the other hand, no clear differences between dry or wet AMD serum-exposed cells were appreciated in relation with the distribution of Scribble (Figure 3B).

### Effects of exposure to serum from patients with AMD on scribble expression levels in hRPE cells

The two main Scribble isoforms were detected by western blot in all experimental groups. Serum exposure for 3 days caused a slight increase of the 210 kDa isoform in cells from both control and dry AMD groups, compared with cells of the non-serum group. Interestingly, in wet AMD group cells, the expression was maintained at a similar level as in the non-serum cells (Figures 3C,D). When the exposure time was increased to 7 days, the expression of this protein is maintained at a similar level in all groups, except for a slight decrease in the 5% wet AMD group cells (Figures 3C,D). However, no statistically significant differences in the expression levels of the 210 kDa isoform between the different groups treated with serum were found. On the other hand, we found highly significant differences for the expression of the 175 kDa Scribble isoform among the groups exposed to the serum from different patients (*p* = 0.001; *n* = 143). In contrast to the cells without serum (non-serum) (*n* = 24), this isoform significantly increased its expression level in the cells of the group exposed to control serum at 5% (*n* = 7) (*p* = 0.005) and 10% (*n* = 7) (*p* = 0.009), as well as in the cells exposed to dry AMD serum at 5% (*n* = 7) (*p* = 0.003) and 10% (*n* = 7) (*p* = 0.003); and it is maintained at the same level in the cells exposed to wet AMD serum. As the time of exposure increased up to 7 days, an increment in the expression of this isoform was observed in the 10% dry AMD (*n* = 6) (*p* = 0.043) and 10% wet AMD (*n* = 10) (*p* = 0.002) groups compared with cells that were not exposed to sera. Thus, a very different impact on the expression level of Scribble was evident in hRPE cells depending on the exposure to serum from patients with diverse AMD pathological backgrounds.

## Discussion

The RPE is a simple epithelial tissue located in the interface between the neural retina and the choroid, and constitutes the outer blood-retinal barrier of the retina (Strauss, 2005). It is defined by a pronounced apicobasal polarity and robust tight junctions, providing the basis for all the functions it performs (Bok, 1993; Rizzolo, 1997, 2007; Rahner et al., 2004; Strauss, 2005; Lehmann et al., 2014). Apicobasal polarity is an inherent feature in many tissues, which must be perfectly orchestrated (Assémat et al., 2008; Kaplan et al., 2009), and when disrupted, it triggers multiple signaling pathways and molecular processes that ultimately lead to pathology development (Stein, 2002; Mostov et al., 2003; Coradini et al., 2011). In epithelial tissues, cell polarity is established by the coordinated and cooperative action of three evolutionary, highly conserved polarity complexes known as Par, Crb, and Scribble. Apical polarity complexes Par and Crb have been described in the human retina and RPE (van de Pavert et al., 2004; Luo et al., 2006; Park et al., 2011; Paniagua et al., 2015, 2021), but the expression and localization of the Scribble complex remained largely unexplored in this tissue.

Scribble complex in mammals is composed of three proteins: Scribble, DLG, and LLGL, and it is associated to the basolateral domain of the epithelial cell membrane, overlapping adherens junctions (Qin et al., 2005; Kallay et al., 2006). It function cooperatively to establish and regulate cell polarity, junction formation, cell growth, and migration in most epithelial cells (Kallay et al., 2006; Yamanaka and Ohno, 2008; Su et al., 2012).

In the present work, we have defined, for the first time, the precise localization and the expression pattern of Scribble in human RPE cells *in vitro*. Results obtained by Western blot and immunofluorescence during the differentiation process in cultured cells have allowed us to establish the presence of Scribble in human RPE cells as early as 7 DIC, when it was found close to the basal membrane in only a few cells. As RPE tissue reached a mature status (Paniagua et al., 2021), its location changed, acquiring a well-defined location on the basolateral membrane of fully polarized mature RPE cells at 21 DIC. Also, there is much controversy in the literature, as to which is the more characteristic and functional isoform of the Scribble protein since some authors have focused on the 210 kDa isoform (Vieira et al., 2008; Phua et al., 2009; Boczonadi et al., 2014), while others have centered their efforts on the 175 kDa one (Métais et al., 2005; Assémat et al., 2008; Su et al., 2013). Since its function is largely unknown in the RPE, we decided to analyze the expression of the two isoforms in this tissue under different conditions, such as development, and under pathological-simulated conditions to shed light on this issue.

Although Scribble is expressed in a multitude of mammalian cell types and tissues, the highest expression levels have been found in epithelial cells, where it plays a coordinating role together with apical polarity complexes in establishing and regulating apicobasal polarity, junction formation, cell growth, or migration (Navarro et al., 2005; Kallay et al., 2006; Assémat et al., 2008; Yamanaka and Ohno, 2008; Su et al., 2013). In 2005, it was described for the first time the widespread protein expression of Dlg-1 and Scribble in the mouse eye during embryonic and postnatal development (Nguyen et al., 2005). Our results have shown that in adult mouse RPE cells, Scribble localization is restricted to the basolateral domain, as double immunofluorescence labeling for Scribble, and the proteins known to be in the apical side demonstrated that they never colocalized in these cells. Additionally, we have confirmed that the distribution of this protein in the basal domain of the mouse RPE cells is continuous and uniform throughout the cell surface. Other works have shown that high concentrations of Dlg-1 and Scribble seem to be localized at regions known to contain apical junctional complexes in several parts of the eye, in which they often overlap with E- cadherin, N-cadherin, or ZO-1, suggesting they may play different roles in cell adhesion and differentiation. Intriguingly, the distribution of this protein in RPE seems to differ. It has been shown that Scribble colocalizes with Dlg-1, like in most ocular structures, and E-cadherin colocalized with Dlg-1 but not with Scribble, as occurs in the corneal or the lens epithelia. Interestingly, although Scribble colocalizes with ZO-1 in most eye structures, there was only minimal colocalization of the two proteins on the apical surface in this case, and Scribble was also distributed along the basolateral sides of the cells. While the punctate labeling on the apical surface suggested some overlap of these proteins in the tight junctions in RPE, the authors suggested that Scribble could be involved not only in the establishment of tight junctions but also in the organization of cell adhesion complexes in the RPE. This was the first time it was suggested that Dlg and Scribble may not only have functions that are conserved across species, but also may be additionally involved in the regulation of cell adhesion, growth and differentiation in mammalian organ systems, such as the eye (Nguyen et al., 2005). Later, it was found that all the three proteins of the Scribble complex were widely distributed through the adult mouse retina (Vieira et al., 2008). When studying a transgenic mouse, which developed tumors in the RPE, they observed changes in the behavior of these proteins during ocular carcinogenesis, including their mislocalization and downregulation (Vieira et al., 2008). Both the mislocalization and downregulation of Dlg-1, Scribble, and Lgl1 proteins seemed to be correlated to tumor progression in the RPE, due to their role in the onset of epithelial-to-mesenchymal transition (EMT), which was later found to be a common event in more aggressive human tumors (Gardiol et al., 2006; Humbert et al., 2008; Pearson et al., 2011; Elsum et al., 2012; Stephens et al., 2018), which also occurs in age-related macular degeneration (AMD) development (Goldberg et al., 2018). Alterations in Dlg1, Scribble, and Lgl1 functions could promote tumorigenesis by, first, promoting hyperplasia and, second, leading to a loss of cell polarity, thereby reducing cell adhesion and facilitating aggressive overgrowth and invasive behavior (Vieira et al., 2008), suggesting they are involved in pathways that play a major role in cancer development (Zhan et al., 2008; Elsum et al., 2012; Feigin et al., 2014; Pearson et al., 2015).

Age-related macular degeneration (AMD) is a chronic, multifactorial, and highly disabling neurodegenerative pathology of the retina that mainly affects people over 50 years of age (Mitchell et al., 2018). The RPE is located at the core of AMD pathogenesis (Bhutto and Lutty, 2012; Ferrington et al., 2016; Datta et al., 2017). The RPE is damaged in the early stages of pathology, and due to its strategic location, RPE injury affects the choroid and photoreceptors, causing them to degenerate, and, ultimately, leading to blindness (Ach et al., 2015; Gupta et al., 2017; Tarau et al., 2019). Disruption of the epithelial barrier and tight junctions' disassembly is well-documented in AMD. The integrity of tight junctions is necessary for maintenance of cell polarity, whereas correct segregation of the apical and basolateral plasma membrane domains mediated by different protein polarity complexes is essential for their structure and stability (Knust, 2002; Shin et al., 2006). There are different works where blood serum collected directly from patients suffering from different diseases has been used to simulate *in vitro* certain pathological conditions occurring *in vivo* (Minagar et al., 2003; Sattler et al., 2019; Curtaz et al., 2020). In fact, a modified commercial human serum has also been used to establish a controlled environment that mimics the AMD-like scenario, promoting drusen formation (Johnson et al., 2011), where 10% of human sera was stablished as a preferred concentration to mimic this condition. In the pathological hRPE model used in this work, where cells are exposed to serum obtained from AMD and control patients to generate the experimental conditions that mimic the toxic environment that these cells undergo during the development of AMD, one of the features that indicated anomalies in the cell monolayer was the distribution of F-actin. F-actin cytoskeleton analysis is sufficient to report changes in RPE cultures and identify anomalies in shape and size when these cells are affected (Ach et al., 2015; Müller et al., 2018). The RPE in its normal state has few stress fibers; however, the presence of intracellular stress fibers is a common finding when they are altered (Tarau et al., 2019). In fact, AMD impacts individual RPE cells by cytoskeleton derangement, including separations and breaks around subretinal deposits, thickening, and stress fibers (Ach et al., 2015; Tarau et al., 2019).

Regarding the Scribble complex, it has been linked with the onset and progression of many types of tumors and diseases that primarily result in a loss of polarization and disorganization of the epithelial tissue (Gardiol et al., 2006; Humbert et al., 2008; Pearson et al., 2011; Elsum et al., 2012; Stephens et al., 2018). Scribble was recently identified as a regulator of a transcriptional and signaling pathway of EMT that is involved in tight junction establishment (Elsum et al., 2013) and is also important for tight junction assembly and function in intestinal epithelial cells (Ivanov et al., 2010). Immunofluorescence analysis of Scribble location in serum-exposed cells shows a disorganization of its distribution, which is common in cells exposed to both control and serum from patients with AMD, although a few cells partially preserve its expression at the plasma membrane. Scribble disruption occurs independently from the exposure time or serum concentration employed. Western blot analysis showed increased expression only of the 175 kDa isoform of Scribble after exposure to serum, especially with control and dry AMD serum. After 7 days with serum, the increase was more evident as the cells were exposed to the highest concentration of AMD serum used in this work. It has been shown that, under inflammatory conditions, Scribble acquires an abnormal distribution in the cytoplasm in epithelial cells both *in vitro* and *in vivo* (Ivanov et al., 2010), as it occurs in this hRPE model following the serum exposure. Prior studies in other cell models have shown that mislocalization of the protein or its loss leads to polarity alteration in human epithelial cells, leading to dysregulation of their function (Zhan et al., 2008; Pearson et al., 2011; Feigin et al., 2014). Scribble is considered a tumor suppressor, so when its roles are altered, cell proliferation and tissue overgrowth occur, leading to apicobasal polarity and junctional integrity disruption (Qin et al., 2005; Elsum et al., 2013; Pearson et al., 2015; Saito et al., 2018). Therefore, these data suggest that the Scribble delocalization from the plasma membrane that was observed in our model could be a critical step for the progression of hRPE cells to an EMT process, increasing proliferation and losing apicobasal polarity. Moreover, Scribble is a transcriptional regulator of the EMT process, so the delocalization, together with the increased expression of Scribble, might be the result of the activation of this process.

## Conclusion

Apicobasal polarity is an essential feature for the RPE to perform its functions. In this study we have explored both the presence and the alterations of the Scribble protein in this tissue in physiological and pathological conditions. Results obtained have shown for the first time that the polarity protein Scribble is expressed early during the development of this tissue, and that it is precisely located in the basolateral membrane of mature RPE cells soon during this process. Furthermore, we have originally shown that RPE cells exposed to blood serum of both control patients and patients with AMD induces structural changes in the cells, including the cytoskeleton and the Scribble polarity complex, which are reflected on the cell monolayer integrity, especially in cells exposed to the serum of patients with AMD. Alterations in human RPE cells due to AMD serum exposure may be consistent with an epithelial-to-mesenchymal transition process, resembling the one that occurs at the beginning of the human disease, although further studies are required to confirm these findings.

## Data availability statement

The raw data supporting the conclusions of this article will be made available by the authors, without undue reservation.

## Ethics statement

The studies involving human participants were reviewed and approved by Clinical Research Ethics Committee of the Institute for Biomedical Research of Salamanca (IBSAL) and the Drug Research Ethics Committee of the Regional Ministry of Health of Junta de Castilla y León. The patients/participants provided their written informed consent to participate in this study. The animal study was reviewed and approved by Bioethics Committee of the University of Salamanca.

## Author contributions

AS and CL conceived the presented idea. AS, AV, and CL designed the methodology and wrote the original draft. EH-G recruited the patients and obtained the blood serum. AS, AR-C, and BC performed, validated, and carried out the formal analysis of the experiments. AV, EH-G, and CL contributed with funding, resources, and supervision. All authors validated the analysis and completed the review, editing of the manuscript, contributed to the final manuscript, and approved the submitted version.

## Funding

This study has been funded by Instituto de Salud Carlos III (ISCIII) through the projects PI15/01240 and PI18/01536 co-funded by the European Union (to CL) and by grants from Consejería de Sanidad de la Junta de Castilla y León (GRS2334/A/21 and GRS2167/1/2020). AS was supported by a pre-doctoral fellowship from Junta de Castilla y León co-financed by the European Social Fund.

## Conflict of interest

The authors declare that the research was conducted in the absence of any commercial or financial relationships that could be construed as a potential conflict of interest.

## Publisher's note

All claims expressed in this article are solely those of the authors and do not necessarily represent those of their affiliated organizations, or those of the publisher, the editors and the reviewers. Any product that may be evaluated in this article, or claim that may be made by its manufacturer, is not guaranteed or endorsed by the publisher.
