# Supplementary material for: Scribble basal polarity acquisition in RPE cells and its mislocalization in a pathological AMD-like model
Source: Front Neuroanat. 2022 Sep 23;16:983151. doi: 10.3389/fnana.2022.983151 (PMC9539273; doi:10.3389/fnana.2022.983151)
Supplement: Supplementary file 1 [file Data_Sheet_1.PDF]

Supplementary material

1. Statistical analysis of Scribble protein expression during the development and establishment of the hRPE cells in culture (in Figure 1):

Descriptives

		N	Mean	Std. Deviation	Std. Error	95% Confidence Interval for Mean		Minimum	Maximum
						Lower Bound	Upper Bound		
Total_ Scribble	7 DIC	9	1,000000	0E-7	0E-7	1,000000	1,000000	1,0000	1,0000
	14 DIC	9	1,006556	,1807434	,0602478	,867624	1,145487	,6550	1,2097
	21 DIC	9	1,068400	,4004614	,1334871	,760578	1,376222	,3826	1,7267
	Total	27	1,024985	,2457280	,0472904	,927778	1,122192	,3826	1,7267
Scribble_ 210kDa	7 DIC	9	1,000000	0E-7	0E-7	1,000000	1,000000	1,0000	1,0000
	14 DIC	9	,860122	,1507034	,0502345	,744281	,975963	,6043	1,1339
	21 DIC	9	,938144	,5175062	,1725021	,540354	1,335935	,3862	1,9575
	Total	27	,932756	,3046203	,0586242	,812252	1,053259	,3862	1,9575
Scribble_ 175kDa	7 DIC	9	1,000000	0E-7	0E-7	1,000000	1,000000	1,0000	1,0000
	14 DIC	9	1,152933	,3002709	,1000903	,922125	1,383742	,5310	1,5484
	21 DIC	9	1,198622	,3717484	,1239161	,912871	1,484373	,2724	1,4959
	Total	27	1,117185	,2788477	,0536643	1,006877	1,227494	,2724	1,5484

First, normality and homoscedasticity were tested:

Tests of Normality

	Kolmogorov-Smirnov[a]			Shapiro-Wilk		
	Statistic	df	Sig.	Statistic	df	Sig.
Total_Scribble	,197	27	,009	,925	27	,053
Scribble_210kDa	,227	27	,001	,860	27	,002
Scribble_175kDa	,226	27	,001	,890	27	,008

a. Lilliefors Significance Correction

Test of Homogeneity of Variances

	Levene Statistic	df1	df2	Sig.
Total_Scribble	12,338	2	24	,000
Scribble_210kDa	13,464	2	24	,000
Scribble_175kDa	4,115	2	24	,029

Based on these data we were able to conclude that the population did NOT follow a normal distribution and did NOT present homoscedasticity, so we decided to apply non-parametric tests. Since we wanted to make comparisons between the three different groups (7, 14 and 21

DIC) we used the Kruskal-Wallis test for multiple pairwise comparisons. The following significance values were obtained, and we were able to conclude that there were only significant differences in the expression of the 175 kDa Scribble isoform.

Hypothesis Test Summary

	Null Hypothesis	Test	Sig.	Decision
1	The distribution of Total_Scribble is the same across categories of Days_in_culture.	Independent-Samples Kruskal-Wallis Test	,828	Retain the null hypothesis.
2	The distribution of Scribble_210kDa is the same across categories of Days_in_culture.	Independent-Samples Kruskal-Wallis Test	,112	Retain the null hypothesis.
3	The distribution of Scribble_175kDa is the same across categories of Days_in_culture.	Independent-Samples Kruskal-Wallis Test	,015	Reject the null hypothesis.

Asymptotic significances are displayed. The significance level is ,05.

SCRIBBLE 175 kDa

Independent-Samples Kruskal-Wallis Test

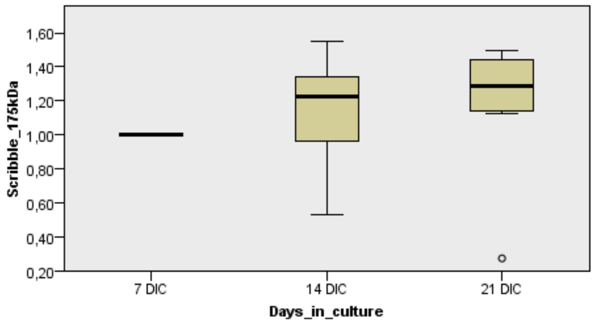

**Total N**	27
**Test Statistic**	8,451
**Degrees of Freedom**	2
**Asymptotic Sig. (2-sided test)**	,015

1. The test statistic is adjusted for ties.

Pairwise Comparisons of Days_in_culture

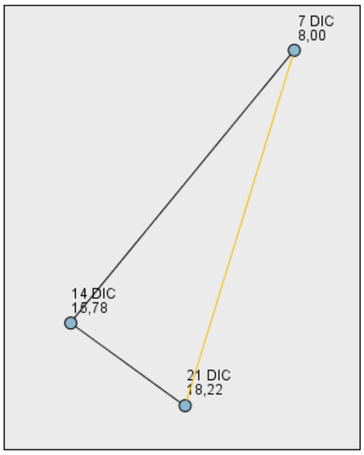

Each node shows the sample average rank of Days_in_culture.

Sample1-Sample2	Test Statistic	Std. Error	Std. Test Statistic	Sig.	Adj.Sig.
7 DIC-21 DIC	-10,222	3,672	-2,783	,005	,016
7 DIC-14 DIC	-7,778	3,672	-2,118	,034	,103
14 DIC-21 DIC	-2,444	3,672	-,666	,506	1,000

Each row tests the null hypothesis that the Sample 1 and Sample 2 distributions are the same.
Asymptotic significances (2-sided tests) are displayed. The significance level is ,05.

When comparing groups 2 to 2 in this case, it was observed that the significant differences were due to the comparison between 7 and 21 DIC.

2. Statistical analysis of Scribble protein expression in hRPE cells after exposure to blood serum from ophthalmic patients (in Figure 3):

Descriptives

		N	Mean	Std. Deviation	Std. Error	95% Confidence Interval for Mean		Minimum	Maximum
						Lower Bound	Upper Bound		
Scribble 210 kDa	Non-serum (24 Days)	24	1,0000	,00000	,00000	1,0000	1,0000	1,00	1,00
	5% Control 3DWS	7	1,4027	,32425	,12255	1,1028	1,7025	,91	1,94
	10% Control 3DWS	7	1,2795	,35178	,13296	,9541	1,6048	,70	1,78
	5% Dry AMD 3DWS	7	1,4180	,38246	,14456	1,0643	1,7717	,83	2,05
	10% Dry AMD 3DWS	7	1,3372	,40622	,15354	,9615	1,7129	,97	1,93
	5% Wet AMD 3DWS	10	1,2108	,31793	,10054	,9834	1,4382	,66	1,65
	10% Wet AMD 3DWS	10	1,2395	,42347	,13391	,9366	1,5424	,67	2,05
	Non-serum (28 Days)	24	1,0000	,00000	,00000	1,0000	1,0000	1,00	1,00
	5% Control 7DCS	7	1,0005	,54370	,20550	,4976	1,5033	,59	2,17
	10% Control 7DCS	7	1,0798	,68634	,25941	,4450	1,7145	,44	2,56
	5% Dry AMD 7DWS	7	1,1115	,54697	,20673	,6056	1,6173	,55	1,94
	10% Dry AMD 7DWS	7	1,2826	,58495	,22109	,7416	1,8236	,69	1,93
	5% Wet AMD 7DWS	10	,7840	,27130	,08579	,5899	,9781	,34	1,20
	10% Wet AMD 7DWS	10	1,3367	,37906	,11987	1,0656	1,6079	,90	1,98
	**Total**	**144**	**1,1326**	**,37998**	**,03166**	**1,0700**	**1,1952**	**,34**	**2,56**
Scribble 175 kDa	Non-serum (24 Days)	24	1,0000	,00000	,00000	1,0000	1,0000	1,00	1,00
	5% Control 3DWS	7	1,6747	,21479	,08118	1,4761	1,8734	1,30	1,91
	10% Control 3DWS	7	1,6376	,28897	,10922	1,3703	1,9048	1,28	1,99
	5% Dry AMD 3DWS	7	1,7642	,37921	,14333	1,4135	2,1149	1,16	2,29
	10% Dry AMD 3DWS	7	1,7334	,42495	,16062	1,3404	2,1265	1,33	2,44
	5% Wet AMD 3DWS	10	1,2448	,34854	,11022	,9954	1,4941	,65	1,78
	10% Wet AMD 3DWS	10	1,3135	,32045	,10133	1,0843	1,5427	,81	2,09
	Non-serum (28 Days)	24	1,0000	,00000	,00000	1,0000	1,0000	1,00	1,00
	5% Control 7DCS	7	1,2385	,53600	,20259	,7428	1,7342	,56	2,23
	10% Control 7DCS	7	1,6645	1,20977	,45725	,5456	2,7833	,58	4,18
	5% Dry AMD 7DWS	7	1,4164	,42405	,16028	1,0242	1,8085	,96	2,01
	10% Dry AMD 7DWS	6	2,3053	1,27374	,52000	,9686	3,6420	,92	4,35
	5% Wet AMD 7DWS	10	1,1016	,37195	,11762	,8355	1,3676	,63	1,97
	10% Wet AMD 7DWS	10	1,7724	,56577	,17891	1,3677	2,1772	,96	2,43
	**Total**	**143**	**1,3571**	**,57406**	**,04801**	**1,2622**	**1,4520**	**,56**	**4,35**

First, normality and homoscedasticity were tested:

One-Sample Kolmogorov-Smirnov Test

		Scribble 210 kDa	Scribble 175 kDa
N		144	143
Normal Parameters[a,b]	Mean	1,1326	1,3571
	Std. Deviation	,37998	,57406
Most Extreme Differences	Absolute	,236	,177
	Positive	,236	,177
	Negative	-,104	-,162
Kolmogorov-Smirnov Z		2,836	2,113
Asymp. Sig. (2-tailed)		,000	,000

a. Test distribution is Normal.

b. Calculated from data.

Test of Homogeneity of Variances

	Levene Statistic	df1	df2	Sig.
Scribble 210 kDa	8,005	13	130	,000
Scribble 175 kDa	9,415	13	129	,000

As the population was NON-normal and NON-homoscedastic, parametric tests were applied. The Kruskal-Wallis test was performed for pairwise multiple comparisons. This test showed that there were significant differences between the groups, so we analyzed between which groups these differences were observed.

Hypothesis Test Summary

	Null Hypothesis	Test	Sig.	Decision
1	The distribution of Scribble 210 kDa is the same across categories of Experiment.	Independent-Samples Kruskal-Wallis Test	,001	Reject the null hypothesis.
2	The distribution of Scribble 175 kDa is the same across categories of Experiment.	Independent-Samples Kruskal-Wallis Test	,000	Reject the null hypothesis.

Asymptotic significances are displayed. The significance level is ,05.

SCRIBBLE 210 kDa

Independent-Samples Kruskal-Wallis Test

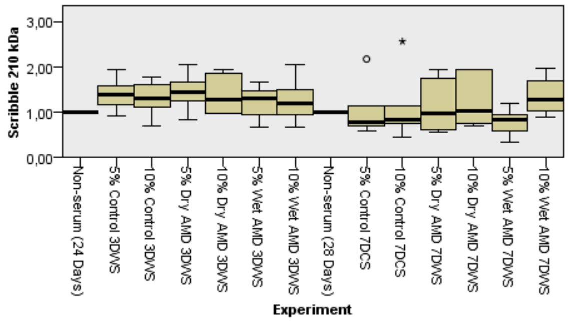

**Total N**	144
**Test Statistic**	36,178
**Degrees of Freedom**	13
**Asymptotic Sig. (2-sided test)**	,001

1. The test statistic is adjusted for ties.

Pairwise Comparisons of Experiment

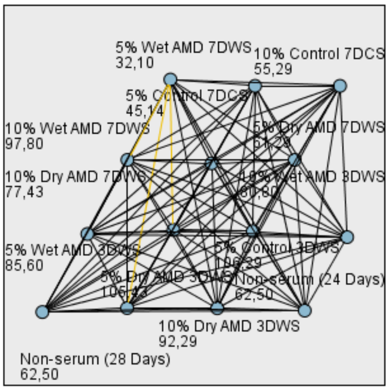

Each node shows the sample average rank of Experiment.

Sample1-Sample2	Test Statistic	Std. Error	Std. Test Statistic	Sig.	Adj.Sig.
5% Wet AMD 7DWS-5% Control 3DWS	74,186	20,172	3,678	,000	,021
5% Wet AMD 7DWS-5% Dry AMD 3DWS	73,329	20,172	3,635	,000	,025
5% Wet AMD 7DWS-10% Wet AMD 7DWS	-65,700	18,306	-3,589	,000	,030

Although the Kruskal-Wallis test result reported significant differences in Scribble-210 kDa expression between groups, the differences yielded by the test were of no interest in our study.

SCRIBBLE 175 kDa

As for the expression of Scribble-175 kDa, we did observe significant differences that were of interest for the present study.

Independent-Samples Kruskal-Wallis Test

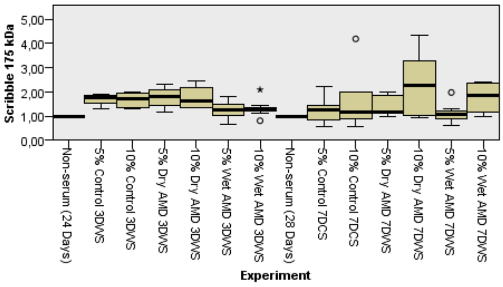

Total N	143
Test Statistic	69,742
Degrees of Freedom	13
Asymptotic Sig. (2-sided test)	,000

1. The test statistic is adjusted for ties.

Pairwise Comparisons of Experiment

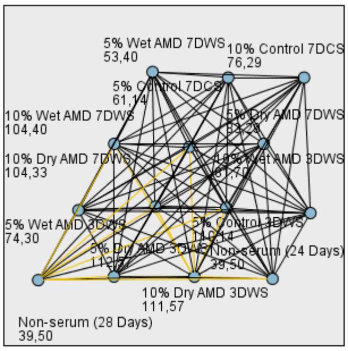

Each node shows the sample average rank of Experiment.

Sample1-Sample2	Test Statistic	Std. Error	Std. Test Statistic	Sig.	Adj.Sig.
Non-serum (28 Days)-10% Wet AMD 7DWS	-64,900	15,294	-4,243	,000	,002
Non-serum (24 Days)-10% Wet AMD 7DWS	-64,900	15,294	-4,243	,000	,002
Non-serum (28 Days)-10% Dry AMD 3DWS	72,071	17,455	4,129	,000	,003
Non-serum (24 Days)-10% Dry AMD 3DWS	-72,071	17,455	-4,129	,000	,003
Non-serum (24 Days)-5% Dry AMD 3DWS	-73,071	17,455	-4,186	,000	,003
Non-serum (28 Days)-5% Dry AMD 3DWS	73,071	17,455	4,186	,000	,003
Non-serum (24 Days)-5% Control 3DWS	-70,643	17,455	-4,047	,000	,005
Non-serum (28 Days)-5% Control 3DWS	70,643	17,455	4,047	,000	,005
Non-serum (28 Days)-10% Control 3DWS	67,786	17,455	3,883	,000	,009
Non-serum (24 Days)-10% Control 3DWS	-67,786	17,455	-3,883	,000	,009
Non-serum (28 Days)-10% Dry AMD 7DWS	-64,833	18,547	-3,496	,000	,043
Non-serum (24 Days)-10% Dry AMD 7DWS	-64,833	18,547	-3,496	,000	,043

The differences that we consider relevant in this study are summarized in the following table, in which the pairwise comparison, the p-value obtained and the associated asterisk for graphical representation are represented.

Scribble 175 kDa		*p-value*	*
24 DIC	5% Control 3DCS	.005	**
24 DIC	10% Control 3DCS	.009	**
24 DIC	5% Dry AMD 3DCS	.003	**
24 DIC	10% Dry AMD 3DCS	.003	**
24 DIC	5% Wet AMD 3DCS	1.000	
24 DIC	10% Wet AMD 3DCS	.527	
5% Control 3DCS	10% Control 3DCS	1.000	
5% Control 3DCS	5% Dry AMD 3DCS	1.000	
5% Control 3DCS	5% Wet AMD 3DCS	1.000	
10% Control 3DCS	10% Dry AMD 3DCS	1.000	
10% Control 3DCS	10% Wet AMD 3DCS	1.000	
5% Dry AMD 3DCS	10% Dry AMD 3DCS	1.000	
5% Dry AMD 3DCS	5% Wet AMD 3DCS	1.000	
10% Dry AMD 3DCS	10% Wet AMD 3DCS	1.000	
5% Wet AMD 3DCS	10% Wet AMD 3DCS	1.000	
28 DIC	5% Control 7DCS	1.000	
28 DIC	10% Control 7DCS	1.000	
28 DIC	5% Dry AMD 7DCS	1.000	
28 DIC	10% Dry AMD 7DCS	.043	*
28 DIC	5% Wet AMD 7DCS	1.000	
28 DIC	10% Wet AMD 7DCS	.002	**
5% Control 7DCS	10% Control 7DCS	1.000	
5% Control 7DCS	5% Dry AMD 7DCS	1.000	
5% Control 7DCS	5% Wet AMD 7DCS	1.000	
10% Control 7DCS	10% Dry AMD 7DCS	1.000	
10% Control 7DCS	10% Wet AMD 7DCS	1.000	
5% Dry AMD 7DCS	10% Dry AMD 7DCS	1.000	
5% Dry AMD 7DCS	5% Wet AMD 7DCS	1.000	
10% Dry AMD 7DCS	10% Wet AMD 7DCS	1.000	
5% Wet AMD 7DCS	10% Wet AMD 7DCS	.456	
